# Unraveling the mechanisms of intervertebral disc degeneration: an exploration of the p38 MAPK signaling pathway

**DOI:** 10.3389/fcell.2023.1324561

**Published:** 2024-01-19

**Authors:** Xingmin Zhang, Zilin Zhang, Xiaosong Zou, Yongjie Wang, Jinwei Qi, Song Han, Jingguo Xin, Zhi Zheng, Lin Wei, Tianhui Zhang, Shaokun Zhang

**Affiliations:** ^1^ Department of Spine Surgery, Center of Orthopedics, First Hospital of Jilin University, Changchun, China; ^2^ Jilin Engineering Research Center for Spine and Spinal Cord Injury, Changchun, China

**Keywords:** p38 MAPK pathway, intervertebral disc degeneration (IDD), extracellular matrix (ECM), inflammation, senescence

## Abstract

Intervertebral disc (IVD) degeneration (IDD) is a worldwide spinal degenerative disease. Low back pain (LBP) is frequently caused by a variety of conditions brought on by IDD, including IVD herniation and spinal stenosis, *etc.* These conditions bring substantial physical and psychological pressure and economic burden to patients. IDD is closely tied with the structural or functional changes of the IVD tissue and can be caused by various complex factors like senescence, genetics, and trauma. The IVD dysfunction and structural changes can result from extracellular matrix (ECM) degradation, differentiation, inflammation, oxidative stress, mechanical stress, and senescence of IVD cells. At present, the treatment of IDD is basically to alleviate the symptoms, but not from the pathophysiological changes of IVD. Interestingly, the p38 mitogen-activated protein kinase (p38 MAPK) signaling pathway is involved in many processes of IDD, including inflammation, ECM degradation, apoptosis, senescence, proliferation, oxidative stress, and autophagy. These activities in degenerated IVD tissue are closely relevant to the development trend of IDD. Hence, the p38 MAPK signaling pathway may be a fitting curative target for IDD. In order to better understand the pathophysiological alterations of the intervertebral disc tissue during IDD and offer potential paths for targeted treatments for intervertebral disc degeneration, this article reviews the purpose of the p38 MAPK signaling pathway in IDD.

## 1 Introduction

IDD is a prevalent degenerative disease worldwide. It is considered as the main reason of chronic LBP, which brings severe economic burden and life pressure to society and patients (Andersson, 1999; Katz, 2006; Dagenais et al., 2008; Millecamps et al., 2012; Lin et al., 2018). According to studies, 80% of average people are predicted to experience back discomfort at some point in their lives (Zhang G. Z. et al., 2020). Risk factors for IDD are complex. Heredity, germs, viruses, gender, overweight, smoking, senility, and mechanical load may all contribute to the occurrence of IDD (Dai et al., 2021). From a direct point of view, changes in the IVD’s microenvironment, including cell changes and biochemical environment changes, may lead to structural or functional damage of IVD, which can be an essential factor in the progression of IDD (Wang et al., 2020).

The specific pathological causes of IDD are still unclear. Still, many papers have indicated that excessive inflammatory response, ECM degradation, cell differentiation, aging, apoptosis, oxidative stress and other factors are involved in the progress of IDD, which ultimately result in disc dysfunction or structural damage (Feng et al., 2016; Ao et al., 2019; Ikuno et al., 2019; Zhang G. Z. et al., 2020). The passage of IDD can result in series of diseases, comprising IVD herniation, sciatica, spinal stenosis, *etc.* However, most current treatments focus on relieving symptoms and controlling pain and do not treat from the molecular level of IDD pathogenesis (Le Maitre et al., 2015). The study of cell biology has allowed us to treat the IVD on a microscopic level in order to postpone or hinder intervertebral disc degeneration (Shimer et al., 2004; Walsh et al., 2004; Masuda and An, 2006). Hence, in order to create efficient, secure treatments, it is critical to comprehend the intricate mechanisms causing IDD.

**FIGURE 1:**
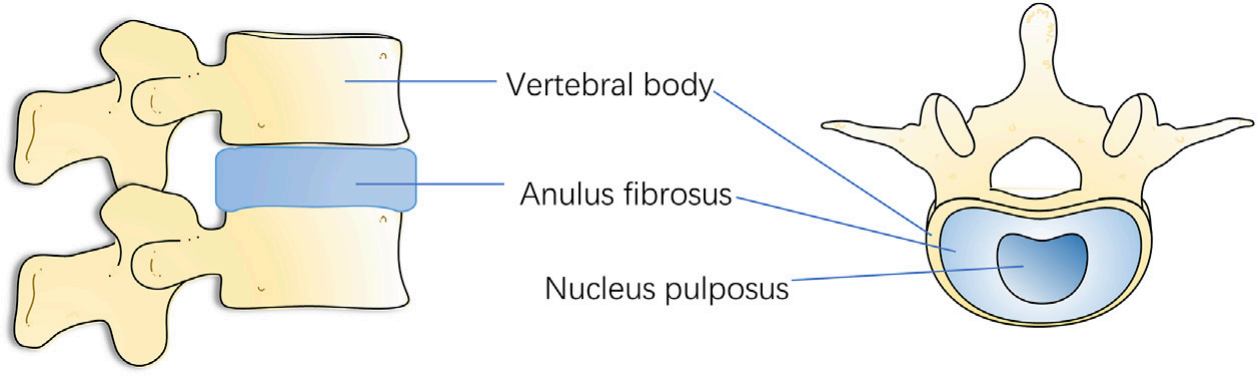
Lateral view and top view of the vertebra and disc.

Spinal vertebrae are connected by a fibrocartilaginous tendon called intervertebral disc. It supports the spine’s load and offers mobility and flexibility (Liang et al., 2014). It has also led to the fact that IVD may be the first degenerative change tissue in human body (Rider et al., 2019). Normal IVD is made up of a central gelatinous nucleus pulposus (NP), annulus fibrosus (AF) surrounding the NP, and a cartilaginous endplate (CEP) connecting the upper and lower vertebral bodies (Guo et al., 2020). The gelatinous NP in the center consists of collagen type II (COL2), glycosaminoglycan (GAG), and NP cells (NPCs), while the surrounding fibrocartilage is mainly composed of collagen type I fibers and AF cells. The upper and lower CEPs are similar to hyaline cartilage and seal the disc (Bowles and Setton, 2017) (Figure 1). When IDD occurs, the early stages of disc degeneration are the breakdown of proteoglycans followed by the conversion of collagen types. Following the breakdown of aggregated proteoglycans, collagen II is replaced by collagen I, making it less hydrophilic and water retaining. Manifestations such as dehydration of NP, tearing of AF and formation of fissures in CEP occur within the disc tissue, which further develops into ossification of CEP. Narrowing of the channels for substance exchange in the vertebral endplates, which in turn leads to obstruction of the process of exchange of nutrients and metabolites between the disc and the vertebral body, and the collection of a large quantity of acidic substances and matrix degradation products in the disc, which aggravates the blockage of the channels for exchange of substances, thus forming a Vicious cycle, further aggravate the degeneration of AF. Then, the fissure of AF, with the aggravation of the vicious cycle, becomes more and more serious, and the restraining effect of AF is reduced, which will lead to the prolapse of NP bound by AF. with the different degrees of the nucleus pulposus breaking through the fibrous ring, IDD is divided into a total of five grades (Pfirrmann et al., 2001). Many studies have shown that inflammation, oxidative stress, mechanical stress, and other factors promote the degradation of the IVD extracellular matrix, resulting in IVD structural and functional damage (Zhang Y. et al., 2020; Cazzanelli and Wuertz-Kozak, 2020; Wang et al., 2020).

Most cells contain the serine-threonine protein kinase known as mitogen-activated protein kinase (MAPK) (Riquelme et al., 2015). The MAPK signaling pathway can transmit extracellular signals from eukaryotic cells into cells to elicit cellular responses (Tasharrofi and Ghafouri-Fard, 2018). By controlling gene transcription and regulation, it can have an impact on cell growth, differentiation, transformation, and death and is essential for preserving normal cell physiology (Sun et al., 2015). At present, four types of MAPK cascades are known to exist in mammals: ERK, JNK/stress-activated protein kinase, p38 MAPK, and ERK5 signal transduction pathways (Jiang et al., 2020). The p38 MAPK signaling pathway, as an essential member of the MAPK signaling pathway family, is involved in the regulation of multiple processes of IDD. Signals from p38 MAPK serve crucial regulatory functions in cell proliferation, apoptosis, autophagy, and differentiation as well as being essential for the catabolic and anti-anabolic impacts of numerous cytokines (Sobajima et al., 2005). Hence, the p38 MAPK signaling pathway is discussed in this article along with how it relates to IDD.

## 2 The pathogenic mechanism of IDD

IDD is not an isolated spinal disorder, but a complex disease caused by multiple factors. Risk factors for IDD include genetics, bacteria, viruses, sex, obesity, smoking, aging, and mechanical loading (Dai et al., 2021). In addition, some of the degenerative changes that take place in the disc as disc degeneration occurs, including narrowing of the intervertebral space, dysfunction of NPCs, Malnutrition, disruption of AF, and calcification of the vertebral endplates (Le Maitre et al., 2005). During these processes, inflammatory response, deterioration of ECM, cellular senescence, oxidative stress response, cell proliferation inhibition, cell autophagy, apoptosis, cell differentiation and cell migration all contribute to the progression of IDD.

### 2.1 Inflammation

Inflammation is one of the main factors contributing to IDD. Pro-inflammatory molecules were found in much higher concentrations in the degenerated intervertebral disc tissue, indicating that inflammation and IDD are intimately connected (Wang et al., 2018). More research demonstrates that the release of inflammatory mediators and the recruitment of immune cell infiltration can speed up the development of IDD (Wang et al., 2020). The inflammatory condition of IDD could be present by elevated levels of matrix metalloproteinases (MMPs), IL-1β, TNF-α, nitric oxide, IL-6, IL-17, IL-9, and prostaglandin E2 (PGE2), and a decrease in anti-inflammatory molecules (Das, 2019). High levels of expression of these inflammatory factors can stimulate the sinus spinal nerves and cause chronic pain in patients due to disc degeneration (Zhang G. Z. et al., 2020). In addition, the expression of inflammatory factors may cause ECM degradation and cellular senescence and apoptosis (Wuertz and Haglund, 2013; Risbud and Shapiro, 2014; Johnson et al., 2015). These effects of inflammatory factors lead to the further development of IDD.

### 2.2 Deterioration of ECM

The balance between synthesis and catabolism of ECM has a very large role in maintaining the homeostasis of the IVD. NPCs are important cells that regulate the synthesis of ECM. The properties of the matrix produced in degenerated NP differed from those of normal NP, with a shift from collagen II to collagen I, and the synthesis of proteoglycans from aggregated proteoglycan to multifunctional glycan, disaccharide proteoglycan, and core proteoglycan (Qi et al., 2019). When the synthetic function of NP cells is disrupted, the synthetic capacity of ECM is reduced. As mentioned earlier, NP cells are mainly composed of collagen II, GAG, and NPCs, while AF is mainly composed of collagen I and AF cells. NPCs, AF cells, and CEP cells play an important role in maintaining the stability and normal function of the spine. When the above changes occur, the boundary between NP and AF will become blurred and the carrying capacity for conformity will decrease. In addition, during the IDD, the expression of MMPs and A Disintegrin and Metalloproteinase with Thrombospondin Motifs (ADAMTS) that mediate matrix degradation increases, resulting in increased matrix degradation (Roberts et al., 2000; Weiler et al., 2002; Cui et al., 2010; Vo et al., 2013; Xu et al., 2014). Therefore, when the synthesis of NP is disrupted and the metabolic enzymes in the tissues are elevated, the ECM synthesis in the disc is weaker than the catabolism, which leads to the aggravation of disc degeneration.

### 2.3 Cellular senescence

Over time, the structure and function of the IVD can become damaged due to a variety of molecular or cellular damage. The accumulation of proteins, telomere damage, mitochondrial or DNA damage may lead to cellular senescence (Finkel and Holbrook, 2000; Hasty et al., 2003; Guarente, 2008; Niedernhofer and Robbins, 2008). Cellular senescence is a cellular state, a stagnation of cellular proliferation caused by some stimuli, a loss of proliferative capacity of normal cells (Campisi, 2005). Cell cycle blocking protein p16^INK4a^ and senescence-associated β-galactosidase (SA-β-Gal) are considered as reliable cellular senescence markers. Michaloglou, C. et al. (Michaloglou et al., 2005) found that p16^INK4a^ increased in expression with age in patients' IVDs. However, age and proliferating cells in IVD were negatively correlated. It was illustrated in a report that the expression of SA-β-Gal increased with increasing degeneration of disc tissue, but proliferating cells were decreasing in degenerated disc cells (Gruber et al., 2009). This suggests that cellular senescence decreases the proliferation and functionality of cells in the degenerated disc tissue. In addition, senescent cells promote the release of inflammatory factors, triggering processes such as matrix metalloproteinase release and ECM degradation to further aggravate disc degeneration.

### 2.4 Cell apoptosis

Apoptosis, a programmed death, is an important type of IVD cell death and is an important factor in exacerbating IDD (Wang et al., 2014). One article reported the finding that a large proportion of cells among the human degenerative tissue undergo programmed death (Gruber and Hanley, 1998). Apoptosis, or type I programmed death, is characterized by DNA degradation, apoptotic vesicle formation and so on. Apoptosis has been reported to be widespread in diseases such as osteoarthritis, neurodegeneration, and IDD. Persistent cell death may lead to increased progression of IVD (Ding F. et al., 2013). In addition, Excessive cell apoptosis results in decreased ECM synthesis of IVDs and exacerbates IDD. Abnormal apoptosis and accelerated aging of NP cells are considered to be the two main cellular processes associated with IDD.

### 2.5 Cell autophagy

Autophagy is the second type of programmed cell death, characterized by the appearance of autophagosomes, and similar to apoptosis is also widely present in various degenerative diseases. Cellular autophagy eliminates excess cells and also protects cells from stimulation during starvation (Wang et al., 2014). During autophagy, autophagosomal (AP)-dependent lysosomes degrade a variety of cytoplasmic contents, damaged or excess organelles, and abnormal protein aggregates (Cohen-Kaplan et al., 2016; Ruocco et al., 2016). Phagocytic activity was reported to be significantly greater in AF cells that had undergone degenerative changes than in normal cells (Gruber et al., 2011). However, the exact function of autophagy in IDD is less certain, and some articles report a cytoprotective effect of autophagy, especially in the harsh environment of malnutrition. However, there are other reports suggesting that autophagy induces the progression of IDD. Hence, more research is needed to examine the relationship between them.

### 2.6 Oxidative stress response

Oxidative stress, as a survival state of cells, is related to the balance between the generation and degradation rate of ROS (Orrenius, 2007). Oxidative stress is present during the IDD process as well. The generation of ROS is beneficial to IVD cells when conditions are physiological. However, an excessive buildup of ROS will directly harm IVD cells and disturb the IVD matrix’s balance by lowering proteoglycan production and increasing the expression of MMPs, among other effects (Nasto et al., 2013; Hou et al., 2014; Chen et al., 2018). Excessive ROS accumulation induces oxidative stress, which causes functional and structural effects on cells and is closely related to various physiological metabolic processes and signaling networks in intervertebral disc cells within the IVD. In addition, reduced antioxidant activity and elevated concentrations of oxidation products have been reported in degenerated IVD (Feng et al., 2017; Seol et al., 2021). What’s more, Oxidative stress has been shown to cause cartilage destruction in the body by promoting apoptosis, leading to osteoarthritis (Mongkhon et al., 2014; Yu et al., 2014). Therefore, oxidative stress in the IVD may play a very important role in driving the degenerative process of the disc.

## 3 General function and regulation of the MAPK signaling pathway

### 3.1 The structure of the MAPK signaling pathway

**FIGURE 2:**
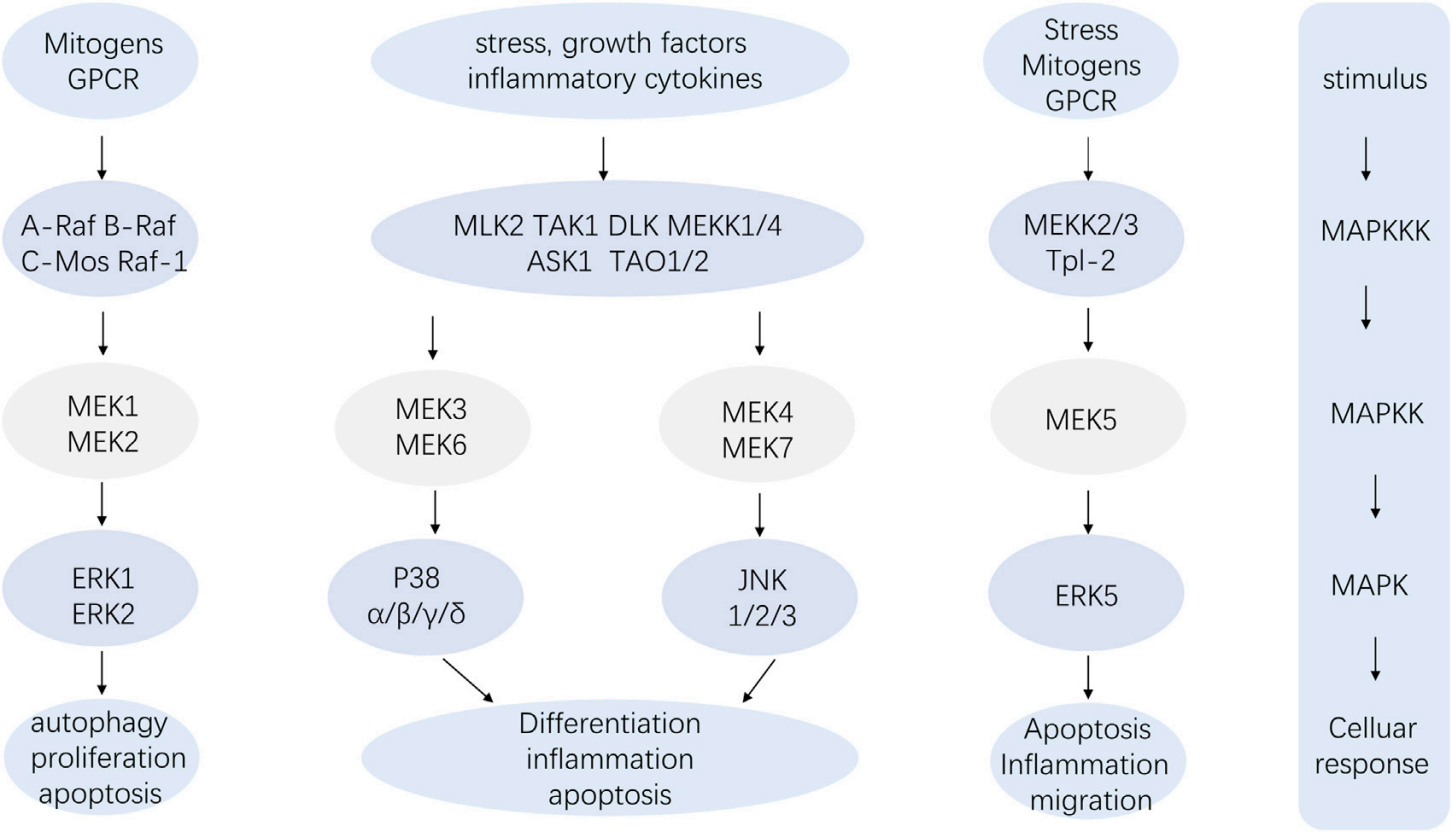
The MAP3K-MAP2K-MAPK chain.

The pathway composition of MAPK is a conserved three-level kinase pattern, including MAPK kinase (MKKK), MAPK kinase (MKK), and MAPK, namely, the MAP3K-MAP2K-MAPK chain (Figure 2). The three kinases can be activated in turn, transmit upstream signals to downstream response molecules, and then participate in various critical cellular physiological and pathological processes such as stress adaptation to the environment and inflammatory response (Wuertz et al., 2012). A range of stimuli, including hormones, growth factors, inflammatory cytokines, peptides acting through G protein-coupled receptors, and environmental risks like ionizing radiation or osmotic pressure, can activate the MAPK signaling pathway (Huang et al., 2010). Similarly, the p38 MAPK module, a component of the MAPK family, also consists of several MAPKKKs, including MEKKs 1 to 4 (MEKK1-4), MLK2 and -3, DLK, ASK1, Tpl2 (also known as Cot) and Tak1, MAPKKs like MEK3, MEK4 and MEK6 (called MKK3, MKK4 and MKK6, respectively) and the four known isoforms of p38 (α, β, γ, and σ) (Roux and Blenis, 2004).

### 3.2 General function of the MAPK signaling pathway

The MAPK signaling pathway exerts a wide range of physiological functions on cells. Among the four branch routes mentioned above, JNK and p38 exhibit similar functions that are associated with inflammation, apoptosis, and growth. ERK primarily participates in cell growth and differentiation. The ERK1/2-MAPK signaling pathway mainly responds to the activation of growth factors, while JNK/MAPK and p38 MAPK are affected by oxidative stress, cytokines, and infection. Firstly, the downregulation of the intensity and duration of the transduction signal regulates the production of proinflammatory cytokines. The inactivation of p38 MAPK and JNK/MAPK pathways stimulates mitogen-activated protein kinase phosphatase-1 (MKP1/DUP1) to weaken tyrosine-like receptor (TLR) signaling in macrophage function and inflammation (Wang et al., 2006). Studies have shown that the MAPK pathway is involved in the activation of NF-κB, which plays a crucial role in the expression of proinflammatory genes such as TNF-α, interleukin, cyclooxygenase-2(COX-2), and inducible nitric oxide synthase (iNOS). Secondly, MAPK signaling pathway plays an important role in cell response to various signals induced by hormones, cytokines and oxidative stress. Apoptosis signal-regulated kinase 1 (ASK1) is a major upstream regulator of MAPK, which is activated under several stressful conditions, including oxidative stress. ROS induces Thioredoxin (Trx) dissociation from ASK1 while TNF receptor associated factor 2 (TRAF2) and TRAF6 are recruited to ASK1 forming an activated state for ASK1. ASk1 phosphorylates MEKS which further phosphorylate JNKs and p38 MAPk leading to their activation. Activated JNKs ansd P38 MAPK induce multiple transcription factor phosphorylation thereby regulating various cellular activities including apoptosis ansd inflammation. Thirdly, Ras acts as an upstream protein for Raf-MEK-ERk pathway being the first small G protein discovered with an active GTP binding conformation along with an ineffective GDP binding conformation, and its active state significantly impacts cell growth and differentiation. It transmits signals by binding MAP3K and Raf kinases to the cell membrane, and then further activates these kinases, thereby regulating cell growth and development. MAPK signaling pathway is the core of signal transduction network involved in regulating cell growth, development and differentiation, and plays an important role in cell proliferation, differentiation, apoptosis and autophagy. More specific effects will be presented below.

## 4 The function of p38 MAPK signaling pathway in IDD

**FIGURE 3:**
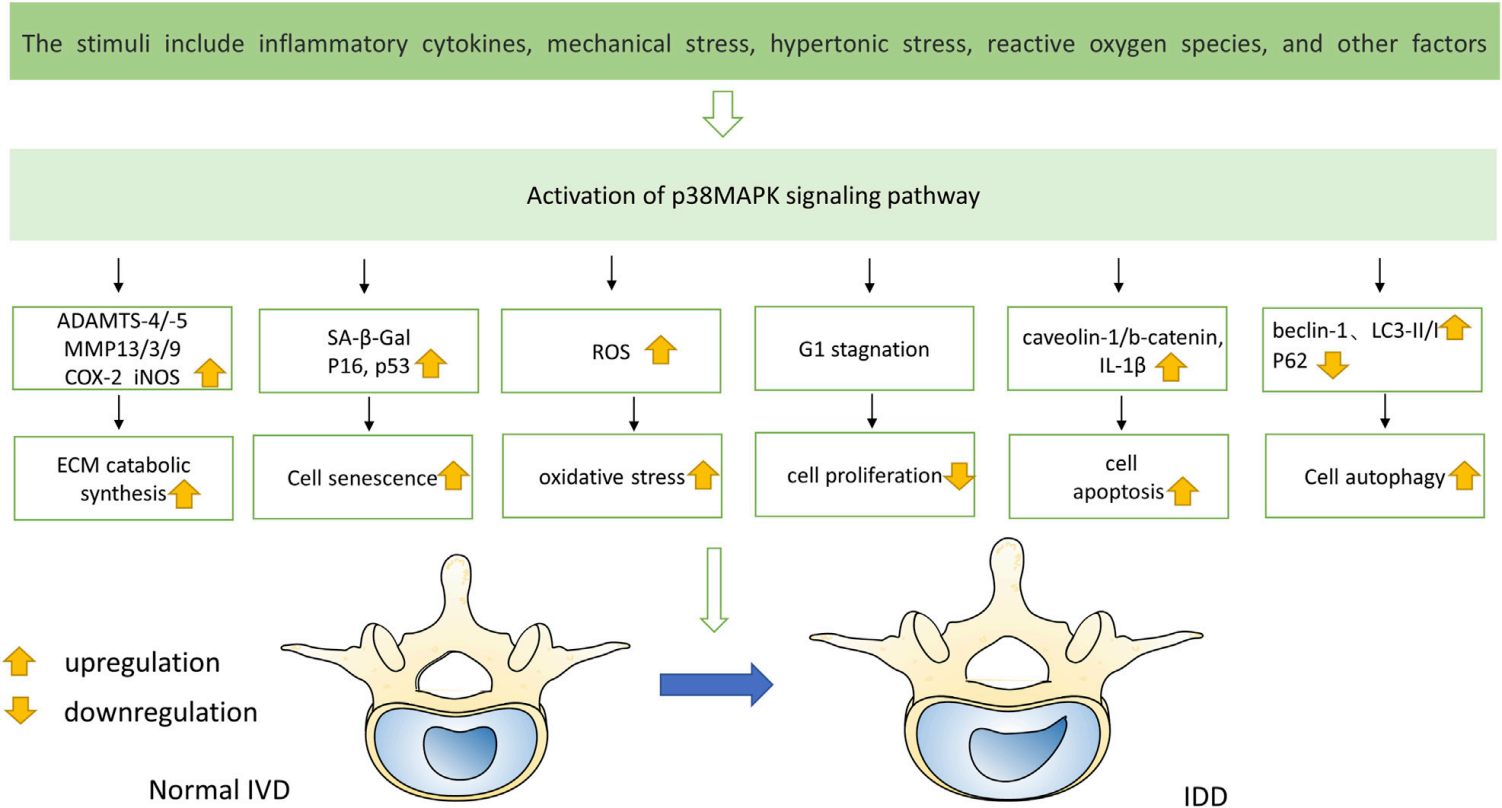
Multiple stimuli lead to the activation of p38 MAPK pathway, resulting in changes of various factors in IVD and eventually disc degeneration.

**FIGURE 4:**
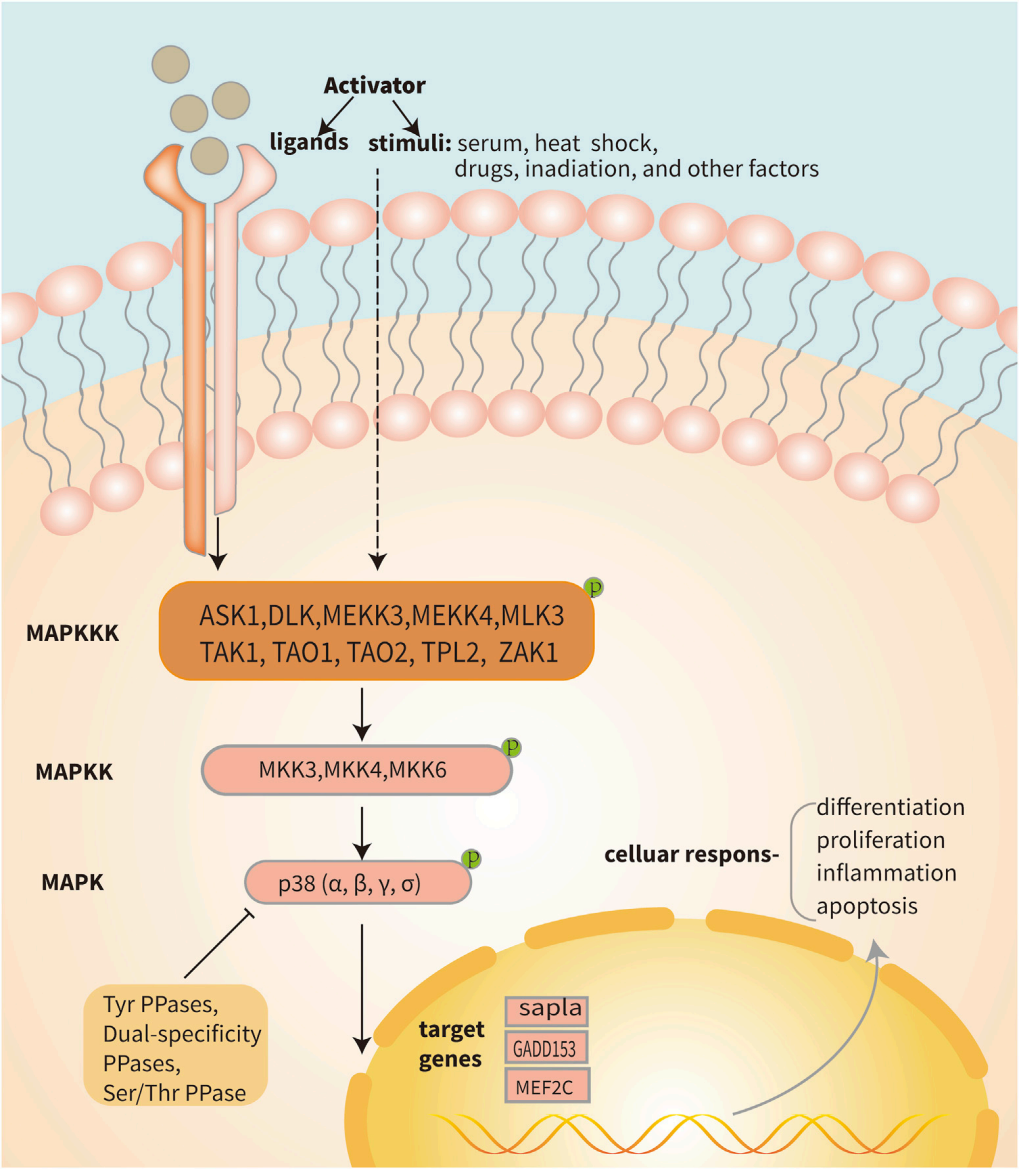
Diagram of the p38 MAPK pathway. Signaling pathways within the cell are activated when the cell is stimulated or when a receptor located on the cell membrane binds to a ligand. The activation of the three-stage cascade of p38 MAPK is initiated by protein signaling, where MAP3Ks such as TAK1, ASK1, and ASK2 phosphorylate MAP2K. Subsequently, activated MEK3/4/6 phosphorylates p38 MAPK, leading to the transcription of its target genes within the nucleus. Ultimately, this triggers a series of cellular changes and reactions. Activated p38 MAPK can be dephosphorylated and thereby deactivated by protein phosphatases, such as dual‐specificity phosphatases, serine/threonine phosphatases and tyrosine phosphatases.

IDD is a complicated illness with multiple risk factors. Interestingly, environmental triggers that are directly related to the pathophysiology of IDD, such as inflammatory chemicals, mechanical stress, and other factors, can activate the p38 MAPK signal transduction pathway (Zhang et al., 2021) (Figure 3). Moreover, compared with the normal tissue, the p38 MAPK phosphorylation is elevated in degenerative IVD tissue, suggesting that IDD may be significantly impacted by the p38 MAPK signaling pathway (Figure 4). The main changes of IDD are ECM deterioration, cell senescence and inflammation. The activation of p38 MAPK can induce inflammation, upregulation of matrix metalloproteinases and other reactions, but also participate in the process of cell senescence, which makes the metabolism of ECM more unbalanced and leads to the deterioration of IVD tissue. According to reports, the p38 MAPK pathway is activated to participate in the development of IDD through a variety of mechanisms and ways (Table 1).

**TABLE 1:** Factors that affect the IDD process by influencing the p38 MAPK pathway.

Factors	Impact on p38 MAPK	Details	References
TNF-α	activate	p38MAPK-dependent upregulation of MMP3 transcripts activation of MAPK kinases including MKK-3 and MKK-6	Mavrogonatou et al. (2014), Zhao et al. (2020)
IL-1β	activate	counteract the catabolic effects of IL-1β	Daniels et al. (2017)
Hypertonic circumstances	activate	inhibit cell growth by causing G2 phase arrest	Mavrogonatou and Kletsas (2009)
high glucose	activate	promote AF cell apoptosis	Shan et al. (2019)
PEMF treatment	activate	inhibit IVD inflammation	Tang et al. (2019)
miR-503	activate	promote NPC apoptosis and degeneration process	Zheng et al. (2020)
IL-17	activate	mediate inflammatory responses in human NPCs in an AP-1-dependent manner	Li et al. (2016a)
Resistin	activate	facilitates macrophage infiltration	Cui et al. (2021)
H_2_O_2_	activate	cause apoptosis and calcification of cartilage endplate cells directly activate ATM downstream targets p53 and p16INK4a, thereby preventing cell proliferation blocking of KMT2D ubiquitination degradation	Han and Sun (2007), Han et al. (2019), Xu et al. (2020)
acidic pH	activate	promote NP senescence	Fu et al. (2018)
high-intensity compression	activate	senescence-related elements up	Fang et al. (2018)
inactivation of FAM20B	activate	spine deformity and disc degeneration associated with AF deformity	Saiyin et al. (2019)
RhTSG-6	inhibit	suppresses IL-1β-induced ECM degradation and apoptosis	Pei et al. (2020)
allicin	inhibit	AOPP-mediated oxidative stress and mitochondrial dysfunction	Xiang et al. (2020)
osteogenic protein-1 (OP-1)	inhibit	reduce NP cell apoptosis and enhance matrix synthesis	Fang et al. (2018)
IL-10	inhibit	elicit anti-inflammatory responses, delaying IDD	Ge et al. (2020)
MALAT1	inhibit	attenuates NPC apoptosis and degeneration process	Zheng et al. (2020)
mesenchymal stem cells	inhibit	alleviate HG-induced ECM degradation	Qi et al. (2019)
Mechano growth factor (MGF) peptide	inhibit	partly reverse apoptosis	Xu et al. (2019)
QUE	inhibit	protected NPCs from apoptosis and prevented ECM degradation	Zhang et al. (2021)
miR-15a, DHJSD (Duhuojisheng decoction)	inhibit	attenuate NPC apoptosis	Cai et al. (2017), Liu et al. (2020)
IAPP	inhibit	increasing the autophagy of NPCs and reducing apoptosis	Wu et al. (2017)
hyperbaric oxygen	inhibit	decreased NO synthesis increased NPCs proteoglycan and collagen II gene expression MMP3, MMP9, and MMP13 was significantly reduced	Niu et al. (2013), Niu et al. (2019)
baicalein	inhibit	inhibit IL-1β-Induced inflammatory response	Jin et al. (2019)
estrogen	inhibit or activate	NP cell matrix is ​consistent with the estrogen function	Li et al. (2016b)

### 4.1 Induce inflammatory response

According to reports, the p38 MAPK drives cytokine-induced inflammation in NPCs and is implicated in several cellular processes linked to IDD (Yang et al., 2016). There are also some studies have reported that p38 MAPKs act as pro-inflammatory mediators and regulate apoptosis and inflammation (Zhu et al., 2020). Other studies have shown that IDD disease is not only a degenerative disorder but also a pro-inflammatory condition.

In particular, one study discovered that by using a p38 MAPK inhibitor in rat NPCs, it is possible to reduce the expression of MMPs and PGE2 in the IVD tissue (Studer et al., 2007). What’s more, in human AF cells co-cultured with macrophages or motivated with TNF-α, Zhang K. et al. (2016) discovered that the p38 MAPK chemical inhibitor SB202190 significantly reduced pro-inflammatory cytokines and prostaglandins, suggesting that p38 MAPK blockage may be beneficial in the therapy of IDD. Studer et al. (2008) also discovered that p38 MAPK inhibition might lessen the induction of PGE2 in human NPCs by IL-1β and TNF-α. Additionally, studies also found a correlation between p38 MAPK and IL-1β and TNF-α. The p38 MAPK can activate signaling molecules that are downstream from it, such as NF-κB and AP-1, and it can also cause the creation of a variety of inflammatory cytokines, like IL-1β, IL-6, and TNF-α (Lv et al., 2021). Moreover, miRNAs can inhibit the p38 MAPK signaling pathway from being activated, which prevents the generation of inflammatory mediators like IL-1β and TNF-α in IVD and delays the development of IDD (Cao and Chen, 2017; Li et al., 2018). In addition, IL-1β and TNF-α can promote the p38 MAPK phosphorylation to activate the p38 MAPK signal pathway. Within 5 minutes of IL-1β-stimulated cell stimulation, Michael B (Ellman et al., 2012). discovered that the PKCδ was rapidly activated. Rapid activation of the p38 MAPK pathways was also observed for >60 min. Other investigations have also demonstrated that IL-1β can activate the p38 MAPK and that lower inhibition of the p38 MAPK probably can counteract the catabolic effects of IL-1β (Daniels et al., 2017). Moreover, When NPCs were treated with hyperbaric oxygen (HBO), decreased IL-1β expression resulted in inhibition of p38 MAPK phosphorylation, decreased NO synthesis, and increased NPCs proteoglycan and collagen II gene expression (Niu et al., 2013). Another study pretreated with baicalein yielded similar results (Jin et al., 2019). As for TNF-α, Eleni Mavrogonatou et al. (Mavrogonatou et al., 2014) indicated that TNF-α caused p38 MAPK-dependent upregulation of MMP3 transcripts in bovine NPCs. TNF-α induced activation of MAPK kinases including MKK-3 and MKK-6 led to the phosphorylation of p38, according to research by ZHAO et al. (Zhao et al., 2020). The aforementioned data hints at the activation of p38 MAPK signaling, which may support the promotion of inflammatory responses during degenerative processes. In a prior study, pulsed electromagnetic fields (PEMF) treatment was reported to reduce IVD cells' expression of IL-6. In a recent study update, it was revealed that the previously noted suppressive impact of PEMF therapy on IVD inflammation is mediated through the signaling pathways of NF-κB and phosphorylated p38 MAPK (Tang et al., 2019). Studies have found that p38 inhibitors can effectively control the production of IL-6 in the inflammatory response (Park et al., 2016). Cui discovered that phosphorylation of p38 increased rapidly after NPCs were treated with visfatin. In addition, NPCs pretreated with p38 inhibitor or targeted siRNA considerably inhibited the expression of IL-6 compared to NPCs treated with visfatin alone (Cui et al., 2021). Inhibition of the p38 MAPK pathway decreased the expression of IL-6 generated by IL-1 and TNF-α in human NPCs, according to Studer RK et al. (Studer et al., 2008). Besides, in another article, Studer et al. (Studer et al., 2007) found that p38 MAPK was associated with increased synthesis of inflammation-and pain-related PGE2 and IL-6 and increased proportion of MMPs. Olga Krupkova et al. (Krupkova et al., 2018) analyzed the gene expression of the endoplasmic reticulum stress marker GRP78 and the pro-inflammatory cytokines IL-6, IL-8, IL-1β, and TNF-α in human surgical IVD samples. They discovered that, in contrast to the other two inflammatory variables, GRP78 was positively linked with the expression of IL-6 in the degree of lumbar degeneration. In another set of cell experiments from surgical samples, endoplasmic reticulum (ER) stress-inducing substances increased IL-6 expression and phosphorylated p38. Both p38 inhibition with SB203580 (10 μM) and CHOP knockdown treatment decreased IL-6 expression in endoplasmic reticulum stress inducer-treated cells. What’s more, in their study, p38 inhibition partially reduced CHOP gene expression, whereas CHOP knockdown did not reduce p38 phosphorylation. Therefore, they concluded that ER stress-induced IL-6 release might be partially dependent on the transcriptional regulation of CHOP by p38.

In addition to these major inflammatory factors, according to several studies, p38 is also implicated in the functional expression of inflammatory factors linked to IL-10, CCL4, and IL-17. Exogenous IL-10 therapy has been shown to suppress p38 MAPK activation and elicit anti-inflammatory responses, delaying the degeneration of IVD (Ge et al., 2020). Through the activation of p38/c-Fos and JNK/c-Jun, it was discovered that IL-17 mediates inflammatory responses in human NPCs in an AP-1-dependent manner (Li J. K. et al., 2016). Resistin binds to TLR4 and enhances CCL4 expression in IVD tissues by activating NF-κB and p38/MAPK, according to the research of Cui’s team (Cui et al., 2021). This facilitates macrophage infiltration. Research has demonstrated that inhibiting p38 in NPCs lowers the synthesis of substances linked to pain, inflammation, and disc catabolism (Studer et al., 2007). The p38 MAPK signaling pathway regulates IVD cells by activating or being activated by upstream and downstream inflammatory factors. Overall, the comprehensive understanding of the role of p38 MAPK in IDD-related inflammation provides valuable insights into potential therapeutic targets for managing disc degeneration and associated inflammatory processes. The findings underscore the significance of p38 MAPK as a key regulator of inflammatory responses and suggest that targeting this pathway may hold promise for developing interventions to mitigate IDD-related inflammation and its detrimental effects on disc health.

### 4.2 Regulate ECM metabolic imbalance

The p38 MAPK is crucial for controlling the production and metabolism of ECM (Seguin et al., 2006; Studer et al., 2008). During IDD, the synthesis function of NP is disrupted, and the metabolic enzymes in the tissue are elevated. The p38 MAPK can affect the expression of MMP1, MMP3, MMP13, IL-6, IL-8, COX-2, iNOS, and TGF-β, as well as control the expression of anabolic and anti-catabolic genes such as proteoglycans, collagen I protein and collagen II, leading to disc degeneration (Zhao et al., 2021). Furthermore, studies have also found that anabolic and anti-catabolic genes encoding aggregation proteins and collagen I and II also seem to be affected by the p38 MAPK signaling pathway (Tu et al., 2017).

During IDD, the p38 MAPK mediates the increased expression of cytokine-induced catabolic enzymes in NPCs, such as ADAMTS-4, -5, and MMPs, which in turn can degrade ECM and lead to aggravation of IDD degree (Tian et al., 2013; Yang et al., 2016). ADAMTS is a recently identified family of metalloproteinases that has been connected to the degradation of aggrecan in IDD. In a recent study, Liu discovered that degenerated disc samples had higher levels of ADAMTS expression and were far more effective at cleaving aggrecan than non-degenerate controls (Liu et al., 2016). What’s more, according to research by Seguin (Seguin et al., 2006), the p38 and ERK signaling pathways influence the synthesis of catabolic enzymes and inflammatory mediators and are involved in proteoglycan metabolism. Additionally, administration of p38 or ERK inhibitors significantly stopped the breakdown of the ECM brought on by cytokines. However, not all p38 MAPK types affect the ECM metabolism. It was found that p38α, p38β, and p38δ isoforms are preferentially expressed in human nucleus pulposus tissues, while the p38γ isoform is absent. The LV-sh-p38α and sh-p38β transfection in NPCs drastically reduced ADAMTS-4, ADAMTS-5, MMP13, and CCL3 expression and restored collagen II and proteoglycans upon IL-1β stimulation, but p38δ played the opposite role (Yang et al., 2016). Therefore, it is mainly p38 α and β that affect the synthesis or catabolism of ECM. Moreover, the inflammatory factors’ expression in NPCs exposed to IL-1 or TNF-α is reported to be reduced in the existence of p38 inhibition, while the proportions of tissue inhibitor of matrix metalloproteinase-1 (TIMP-1) and MMP3 are reported to be raised. It suggested that inhibition of p38 may reduce the degeneration of IVD by inhibiting inflammation and the related IVD matrix breakdown (Studer et al., 2007; Studer et al., 2008).

Numerous studies have discovered that specific stimuli or substances can contribute to the development of IDD by regulating the production of the IVD matrix by blocking or boosting the activity of the p38 MAPK pathway. During the culture of nucleus pulposus mesenchymal stem cells (NPMSCs), high glucose (HG) substantially reduced the expression of aggrecan and collagen II. The p38 MAPK was phosphorylated after exposure to HG, although the level of total p38 MAPK was unaffected. When NPMSCs were treated with MSC conditioned medium (MSC-CM), Qi et al. (Qi et al., 2019) discovered that the phosphorylation of p38 was considerably lower than when the cells were left untreated. The p38 MAPK inhibitor SB203580 was able to reduce the phosphorylation of p38 MAPK and restore Collagen II and proteoglycan expression in NPMSCs. This study demonstrated the potential of MSC-CM to alleviate HG-induced ECM degradation through the p38 MAPK pathway. Moreover, previous research has shown that mechanical stress stimulation causes IDD and that p38 MAPK is involved in that process (Studer et al., 2007; Pang et al., 2017). On this basis, Fang et al. (Fang et al., 2018) found that the p38 MAPK pathway activity was significantly increased under high-intensity compression, and the activity was significantly decreased after adding osteogenic protein-1 (OP-1). In addition, adding OP-1 when culturing NPCs could reduce NP cell apoptosis and enhance matrix synthesis. Hence, they concluded that OP-1 could slow the progression of IDD by increasing matrix synthesis in NPCs by inhibiting p38 MAPK. Additionally, the metabolism of the IVD matrix can be controlled by estrogen’s actions on the p38 MAPK pathway (Li P. et al., 2016). found that estrogen is an upstream molecule of the p38 pathway. When the estrogen function is activated or inhibited, the activity of the p38 MAPK pathway also increases or decreases, and the synthesis trend of the NP cell matrix is ​​also consistent with the estrogen function. Pei et al. (2020) found that Recombinant human TNF-α induced protein 6 (RhTSG-6) suppresses IL-1β-induced ECM degradation and apoptosis by blocking p38 and JNK pathways in NPCs. When NPCs were treated with HBO, the phosphorylation of p38 MAPK, ERK, and JNK was decreased, and the secretion of MMP3, MMP9, and MMP13 was significantly reduced, which reduced the breakdown of matrix by MMPs (Niu et al., 2019). Similarly, Xu et al. found that H_2_O_2_-induced reactive oxygen species (ROS) production promoted the expression of MMP3, MMP9, and MMP13 through p38 MAPK-mediated phosphorylation of KMT2D in NPCs (Xu et al., 2020). Besides, according to some research, IL-1β-induced NO and PGE2 buildup in NPCs could be decreased, and partial proteoglycan synthesis might be restored, by inhibiting the p38 MAPK pathway (Studer et al., 2007; Studer et al., 2008; Li et al., 2015). In summary, according to the results of most studies, the imbalance of ECM metabolism can lead to the rapid deterioration of the microenvironment around the IVD, resulting in accelerated disc degradation, and ECM is directly related to the p38 MAPK signaling pathway. Hence, inhibiting the p38 MAPK pathway may be a significant direction to alleviate the process of IDD.

### 4.3 Accelerate cellular senescence

The senescence of IVD cells is a critical feature of IDD. A crucial assurance for the typical operation of human tissues is the maintenance of a normal cell state. According to recent research, the activity of p38 MAPK pathway can be used as a sign of aging (Wang et al., 2002). Wu (2004), Papadopoulou and Kletsas (2011) found that activation of p38 MAPK via MKK3 or MKK6 could trigger premature senescence by increasing the levels of cyclin-dependent kinase inhibitors. In addition, numerous investigations have discovered that the senescent cells of IVD tissue expressed more p38 MAPK than normal. Gruber et al. (2010) found that upregulation of p38 MAPK gene expression in senescent AF cells in comparison with normal cells by laser capture microdissection (LCM) microarray, which can be used to identify cell-specific gene expression patterns. Fu et al. (2018) also found that acidic pH could promote nucleus pulposus senescence by activating the p38 MAPK pathway. This paper highlights the presence of an acidic ecological environment within the degenerative IVD, thereby impacting the vitality and matrix metabolism of IVD. They placed rat NPCs in a pH 7.2 near-normal environment control group and a pH 6.2 acid environment experimental group for 10 days. They also cultured cells in an acidic environment with the inhibitor SB203580.The results showed that the proliferative capacity of experimental NPCs was inhibited, the G0/G1 phase fraction was increased, while the S phase fraction was decreased, telomerase activity was decreased, the expression of senescence-related molecules (p16 and p53) was upregulated, matrix-related molecules (aggrecan and Collagen II) expression was downregulated, and the p38 MAPK activity was significantly increased. In other experimental NPCs, the inhibitor SB203580 successfully suppressed p38 MAPK activation, lowered the expression of molecules linked to senescence, raised the expression of molecules linked to the matrix, and partially reversed the effects of the acidic niche on the cell cycle. Hence, they came to the conclusion that activation of p38 MAPK can accelerate NP cell senescence. As mentioned above (Studer et al., 2007; Pang et al., 2017), under the influence of mechanical compression stimulation, the p38 MAPK in NPCs can become active. According to (Pang et al., 2017), NPCs under high-intensity compression had much more active p38 MAPK than NPCs under lower pressure, and positive cells stained for senescence-related elements including SA-β-Gal and senescence markers (p16 and p53) had more of them. In the high-pressure culture group added with the inhibitor SB203580, the inhibitor successfully prevented the activation of p38 MAPK pathway and the upregulation of associated aging indicators. Therefore, the above results indicate that the p38 MAPK pathway can promote the senescence of NPCs under intermittent high-intensity compression stimulation. Besides, p38 MAPK can promote cellular senescence through the accumulation of ROS under high-intensity compression (Li P. et al., 2017). found that high-intensity compression resulted in increased p38 MAPK activity and increased ROS production, and also found that p38 MAPK appears to be an upstream effector of ROS and modulates high-intensity compression-induced NP cell senescence, at least in part, through ROS production. Another article found that dehydrocostus lactone (DHE) can reduce the sting signals’ activation and inhibit NF-κB and MAPK pathways to alleviate inflammatory factors to improve the aging of NPCs (Chen et al., 2021). In summary, the passages mentioned above underscore the multifaceted role of p38 MAPK in regulating cellular senescence within IVD tissue. The findings suggest that p38 MAPK activation contributes to premature senescence in IVD cells, particularly under conditions such as acidic pH and high-intensity compression. Targeting the p38 MAPK pathway may hold therapeutic potential for mitigating cellular senescence and its associated detrimental effects on IVD health.

### 4.4 Participate in oxidative stress response

As mentioned earlier, oxidative stress plays a crucial role in the process of IDD. Moderate production of ROS is beneficial for cells, but excessive accumulation of ROS can harm NP cells and cause imbalances in ECM synthesis and catabolism. According to reports, the p38 MAPK pathway is involved in many processes of ROS regulation of IVD tissue (Han et al., 2019). discovered that H_2_O_2_ could cause apoptosis and calcification of cartilage endplate cells through the mitochondrial pathway and ERK/p38/p65 signaling pathway. They also discovered that inhibiting the production of ROS lessens mitochondrial-mediated apoptosis in chondrogenic endplate cells under oxidative stress, which slows the progression of IDD. What’s more, the accumulation of ROS can promote the expression of MMPs. According to reports (Xu et al., 2020), discovered that the human degeneration disc samples have significantly higher levels of methyltransferase KMT2D expression, and the expression of MMPs such as MMP3, MMP9, and MMP13 was significantly downregulated when KMT2D was knocked down with siRNA. They discovered that this mechanism is ROS-induced through KMT2D-mediated H3K4me1 and H3K4me2. Furthermore, they investigated the impact of oxidation-induced KMT2D expression on the upregulation of MMPs. Their findings indicate that H2O2 treatment can impede the ubiquitination degradation of KMT2D, primarily mediated by phosphorylation of p38 kinase. Further studies found that the blocking of KMT2D ubiquitination degradation in response to H_2_O_2_ stimulation was discovered to be reversed by inhibiting p38 MAPK. Therefore, the accumulation of ROS can promote the expression of MMP3, MMP9, and MMP13 through p38 MAPK-mediated phosphorylation of KMT2D in NPCs to disrupt the homeostasis of the intervertebral disc matrix. Besides, several studies have also demonstrated that the presence of oxidative stress and the increase of ROS concentration in IDD can induce the expression of MMP3, MMP9, and MMP13 (Nasto et al., 2013; Dimozi et al., 2015; Tang et al., 2018).

Additionally, it has been discovered in some studies that the p38 MAPK pathway contributes to the catabolic consequences of oxidative stress brought on by elevated ROS levels on AF cells during IDD. In either *in vivo* or *in vitro* tests, the antioxidant N-acetyl cysteine (NAC) effectively reversed the catabolic effects of this ROS (Suzuki et al., 2015). Advanced oxidation protein product (AOPP) is a novel biomarker of oxidative stress, which can disrupt the balance of oxidative stress and result in a number of disorders. Higher AOPP concentrations were discovered in human degenerative IVD, and allicin was found to protect NP cells from AOPP-mediated oxidative stress and mitochondrial dysfunction by inhibiting the p38 MAPK pathway. At the same time, p38 MAPK agonists can relieve this inhibitory effect (Xiang et al., 2020). According to these papers, the p38 MAPK is essential to the oxidative stress response during the development of IDD. It is important that understanding the molecular mechanisms underlying ROS-induced damage and the potential therapeutic implications of targeting the p38 MAPK pathway to mitigate the detrimental effects of oxidative stress in the context of IDD.

### 4.5 Inhibit cell proliferation

The foundation for preserving the IVD tissue’s proper physiological function is its normal proliferative capability. Moreover, the proliferation of IVD cells during IDD also represents the repair capacity of the IVD tissue (Chen et al., 2018). According to certain research, the p38 MAPK pathway can inhibit NPCs’ proliferation and speed up the development of IDD. Han and Sun et al. (Han and Sun, 2007) found that p38 MAPK (also activated by H_2_O_2_) can directly activate ataxia telangiectasia-mutated (ATM) downstream targets p53 and p16^INK4a^, thereby preventing cell proliferation. According to Mavrogonatou and Kletsas et al. (Mavrogonatou and Kletsas, 2009), hypertonic circumstances activate p38, which has inhibitory effects on cell growth by causing G2 phase arrest in bovine NPCs (as seen in IVD during daily activities). Another article discovered that inhibiting the p38 MAPK pathway could partially reverse the high-amplitude compression-induced inhibition of NP cell proliferation. At the same time, they also found that activation of p38 MAPK reduces cell proliferation and telomerase activity, enhances arrest in the G1 phase of the cell cycle, and negatively affects intradiscal cell proliferation (Li P. et al., 2017). Furthermore, other articles discovered that the p38 MAPK pathway is tightly correlated with the process of cell proliferation (Johnson and Lapadat, 2002). found that MAPK signaling pathways, including ERK1/2, JNK, and p38 MAPK pathways, are involved in cell proliferation, migration, and death. Another research article showed that three MAPK family members—ERKs, JNKs, and p38 MAPK—are involved in regulating cell viability and proliferation in NP (Dimozi et al., 2015). Therefore, these studies imply that the p38 MAPK pathway is engaged in the proliferative process of IVD cells. They shed light on the potential implications of p38 MAPK signaling for the repair capacity and physiological function of IVD tissue, offering valuable insights into the molecular mechanisms underlying IDD and potential avenues for therapeutic intervention.

### 4.6 Mediate cell apoptosis

Apoptosis is one of the crucial reasons for IDD. Excessive cell apoptosis results in decreased ECM synthesis of IVDs and exacerbates IDD (Ahsan et al., 2001; Ha et al., 2006; Zhang F. et al., 2016). Studies have revealed a strong connection between p38 MAPK activity and apoptosis (Cuadrado and Nebreda, 2010; Sui et al., 2014). Ariga et al. (2003) found that activation of p38 MAPK regulates mechanical stimulation-induced apoptosis of endplate chondrocytes in mouse IVDs in organ culture. They discovered that as loading weight increased in culture, the number of apoptotic cells increased as well. The number of apoptotic cells was considerably enhanced by two inhibitors, U0126 (MAPK inhibitor) and SB202190 (p38 inhibitor), demonstrating that p38 MAPK can block the apoptosis pathway brought on by mechanical stress. Other studies have shown the importance of MAPK signaling system in the inflammatory-induced death of NPCs, and inhibiting p38 MAPK may be an effective treatment for IDD (Studer et al., 2007). Hiyama A discovered a marked increase in inflammatory cytokines, including IL-1β and TNF-α, in degenerative IVD. Through a series of signaling networks, inflammatory cytokines induce apoptosis in NPCs and lead to progressive IDD (Hiyama et al., 2013). Wang et al. (Wang et al., 2016) found that inflammatory cytokine-induced apoptosis was regulated by caveolin-1/b-catenin signaling by blocking the expression of p38 MAPK, and cytokines controlled caveolin-1/b-catenin signaling via the p38 MAPK pathway. In addition, activation of p38 MAPK also contributes to AF cell apoptosis (Shan et al., 2019). observed the effect of different concentrations of glucose on AF cell apoptosis, and the results showed that high glucose could promote AF cell apoptosis by regulating the JNK pathway and p38 MAPK pathway in a glucose concentration-dependent manner. Numerous studies have demonstrated that a certain substance or factor can affect the p38 MAPK pathway to influence IVD cells' propensity for apoptosis (Jiang et al., 2020). showed that high glucose therapy raised the expression of p38 and p-p38 in rat CEP cells after exposing them to high glucose settings. The expression levels of p38 and p-p38 dropped when the long non-coding RNA MALAT1 (metastasis associated lung adenocarcinoma transcript 1) expression was blocked by MALAT1 RNAi, demonstrating that MALAT1 influences rat CEP cell apoptosis via the p38 MAPK pathway. Similarly, Zheng et al. (2020) investigated the interplay among MALAT1, miR-503, and MAPK pathways. Their study revealed that overexpression of MALAT1 in NPCs led to a reduction in IL-1β upregulated p38, JUN, and Fos levels, while the use of miR-503 mimics or inhibitors resulted in the attenuation or enhancement of their phosphorylation levels. They concluded that MALAT1 acts as a sponge and ceRNA (competing endogenous RNAs) for miR-503 and attenuates IL-1β-induced NPC apoptosis and degeneration process through the MAPK signaling pathway. In another study, In NPCs cultivated under circumstances of mechanical pressure, it was discovered that apoptosis and the activity of the p38 MAPK pathway were markedly elevated. Yet, the p38 MAPK activity was considerably decreased when the Mechano growth factor (MGF) peptide was introduced to the media. The partial reversal of mechanical overload’s effect on apoptosis raises the possibility that MGF can lessen mechanical overload’s ability to cause apoptosis via controlling the p38 MAPK pathway (Xu et al., 2019). Zhang et al. (2021) found that quercetin (QUE) partially inhibited the p38 MAPK signaling pathway and inhibited p38 MAPK through SB203580-activated autophagy, indicating that QUE protected NPCs from apoptosis and prevented ECM degradation through the p38 MAPK autophagy pathway. Besides, RhTSG-6 (Pei et al., 2020), miR-15a (Cai et al., 2017), and DHJSD (Duhuo jisheng decoction) (Liu et al., 2020) have all demonstrated that they could attenuate NP cell apoptosis by inhibiting or blocking the p38 MAPK pathway. Hence, these data imply that the p38 MAPK pathway is also implicated in the promotion of apoptosis. The excessive p38 MAPK activation in IVD cells appears to be a major driver of apoptosis, decreased ECM production, and IDD progression. Inhibiting p38 MAPK phosphorylation through various pathways may help attenuate IVD degeneration by reducing IVD cell apoptosis.

### 4.7 Involve in regulating cell autophagy

Autophagy is a self-degrading function of cells, which can maintain cellular homeostasis and ensure the normal function of cells by degrading or recycling damaged proteins or organelles (Zhang et al., 2021). Studies have found that autophagy is significantly enhanced in degenerative AF and NP compared with normal AF and NP (Jiang et al., 2013; Ye et al., 2013; Kong et al., 2014). Some other studies indicated that the p38 MAPK pathway is engaged in numerous cell activities, including IDD-related apoptosis, autophagy, and other processes (Ariga et al., 2003; Studer et al., 2007). Moreover, some articles suggest that the MAPK pathway is an upstream regulator of autophagy (He et al., 2018; Ba et al., 2019). According to Zhang et al. (2021), QUE increased the expression of autophagy markers beclin-1, LC3-II/I while lowering p62. The protective impact of QUE on apoptosis and ECM deterioration may partially be reversed by the autophagy inhibitor 3-Methyladenine (3-MA), demonstrating that autophagy is implicated in the protective effect of QUE on NPCs. Further research found that QUE partially inhibits the p38 MAPK pathway, and inhibits the p38 MAPK by the inhibitor SB203580 activates autophagy, suggesting that QUE protects NPC from apoptosis and prevents ECM breakdown through the p38 MAPK-autophagy pathway. Hence, they came to the conclusion that QUE was a viable method of treatment for IDD and potentially prevent IDD via modifying p38 MAPK-mediated autophagy. Moreover, Studies have shown that in order to avoid apoptosis and promote autophagy, the p38 and JNK MAPK pathways are often activated in conjunction with the suppression of p38 and JNK protein levels (Chang and Karin, 2001; Hosokawa et al., 2009; Wu et al., 2017) found that the overexpression of islet amyloid polypeptide (IAPP) significantly upregulated the phosphorylation of Akt and mTOR, downregulated the expression of phosphorylated p38 and JNK, and the gene expression of Bcl-2, Beclin1, ATG5 and Atg7, thereby increasing the autophagy of NPCs and reducing apoptosis. When IAPP expression was inhibited with siRNA, PI3K/AKT-mTOR and p38/JNK-MAPK signaling were inhibited, caspase-3 and Bax expressions were increased, and Beclin1, ATG5, and ATG7 expressions were decreased, resulting in enhanced apoptotic effect and reduced autophagic activity in NPCs. These results demonstrate that PI3K/AKT/mTOR and p38/JNK MAPK signaling pathways are involved in the regulation between apoptosis and autophagy by IAPP. At present, there are few studies on the relation between the p38 MAPK and autophagy, therefore further research is required to prove the feasibility of this potential treatment for IDD.

### 4.8 Promote cell migration and cell differentiation

The p38 MAPK is a class of MAPKs that respond to stressful stimuli: cytokines, ultraviolet radiation, heat shock, and osmotic shock, and are involved in processes such as cell differentiation, migration, apoptosis, and autophagy (Das, 2019). Significant roles in the development of IDD are played by cell migration and differentiation. First, in degenerating IVDs, cell migration is influenced by the p38 MAPK pathway. Wang (Wang et al., 2013) discovered that the expression of CCL3, which is higher in degenerating human IVD tissue, is significantly correlated with how quickly tissue degeneration occurs. Their *in vitro* cell migration studies demonstrated that NPCs' produced chemokines promote macrophage migration after TNF-α or IL-1β treatment. The macrophages infiltrate the IVD tissue and lead to degenerative disc disease-related inflammatory responses. They also found the expression of CCL3 is regulated by the inflammatory cytokines TNF-α and IL-1β through MAPK (including p38 MAPK), NF-κB/p65, and C/EBP-β signaling pathways. Similarly (Li Z. et al., 2017), also found a similar role for CCL4. They discovered resistin binds to TLR4 through the p38 MAPK and NF-κB pathways and increases the CCL4’ expression in NPCs, which leads to the infiltration of macrophages. Second, p38 MAPK also promotes the differentiation of various cells in IVD (Yang et al., 2016). discovered that the production of granulocyte macrophage-colony stimulating factor (GM-CSF) and IFNγ triggered by p38α or p38β in NPCs induces macrophage polarization to the M1 phenotype. The role of macrophages in NP may be a normal inflammatory response to tissue damage. They discovered a positive connection between the number of macrophages and the severity of IDD, indicating that macrophages are deleterious to the development of the disease. Therefore, selective targeting of p38 isoforms can reduce macrophage polarization, improve IDD inflammation and alleviate IDD progression. Disorders of the IVD can also be caused by defects in AF (Saiyin et al., 2019). created Fam20B conditional knockout (CKO) mice that had substantial spine deformity and disc degeneration associated with AF deformity. Phosphorylated p38 and phosphorylated ERK were increased, and phosphorylated JNK was decreased in AF of these mice, indicating that inactivation of FAM20B may activate the MAPK-p38/MAPK-ERK pathway in AF. Variations in phosphorylated p38 and ERK levels may cause AF cell differentiation, resulting in disc abnormalities, since changes in the activation of the MAPK signaling pathway may cause AF cells to differentiate into chondrocytes improperly. In addition (Ding W. et al., 2013), discovered that leptin and its receptors are expressed in rat AF cells, and that leptin-induced terminal differentiation of AF cells is probably associated with the upregulation of phosphorylated p38 and ERK1/2, suggesting that p38 MAPK is a valuable player in AF terminal differentiation. Investigations on the function of p38 MAPK in cell migration and cell differentiation are scarce. In summary, the p38 MAPK pathway is a critical mediator in the processes of cell migration and differentiation within the IVD, and its dysregulation contributes to the progression of IDD. Therapeutic strategies targeting this pathway could potentially alleviate the inflammation and cell differentiation disorders that lead to IDD.

## 5 Conclusion and future perspectives

In this review, we summarize pathogenic mechanisms of IDD and findings on the regulation of the p38 MAPK signaling pathway in IDD. During IDD, some external stimuli such as malnutrition, abnormal mechanical loading, and high osmotic pressure induce inflammation, ECM degradation, oxidative stress, programmed cell death, and accelerated cellular senescence to accelerate the progression of IDD. In these processes affecting the development of IDD, p38 MAPK is actively expressed and is involved in the regulation of IDD development by regulating these processes in a direct or indirect manner. Activation of P38 MAPK can increase the expression of inflammatory mediators and matrix degrading enzymes to accelerate inflammation and ECM degradation, a mechanism that can create a vicious cycle leading to increasing IDD. In addition, some signaling molecules upstream and downstream of p38 MAPK can also influence the physiological state within the intervertebral disc such as cellular senescence, cellular autophagy, apoptosis, and proliferation processes by regulating the p38 MAPK pathway. We summarize a variety of drugs and stimuli involved in intervertebral disc regulation by affecting the p38 MAPK pathway in a direct or indirect manner. Although people have some understanding of the p38 MAPK pathway, the specific regulatory targets and mechanisms of the p38 MAPK pathway are still not so clear. For example, we have many literature studies on that one certain drug exerts certain specific effects through the p38 MAPK pathway, but we are not yet clear how this drug exerts its effects through the p38 MAPK pathway. Only by fully understanding the mechanisms and effects throughout the entire process can we better translate the drug into clinical research. Besides, we noticed that some non-coding RNAs and cytokines are essential to the IDD too. Further study of the specific roles of these cytokines and non-coding RNAs will help people understand the molecular pathogenesis of IDD better, and thus may explore new therapeutic ideas. At the same time, while there is some fundamental understanding of the pathway’s role in the IDD process, the extensive involvement of the p38 MAPK pathway in IDD also brings more challenges to treatment. For example, p38 MAPK may act simultaneously in multiple responses related to IDD. How can it work on a specific reaction while attenuating the side effects on the regular physiological activity of IVD cells? What precisely controls the expression changes of p38 MAPK pathway as IDD develops? These more precise and fundamental mechanism studies are clearly more urgently needed in the future, which will provide safer and comprehensive guarantees for p38 MAPK as a therapeutic target for IDD and provide new ideas for future IDD treatment.

## Figures and Tables

**Table:** 

**(IVD)**	Intervertebral disc
**(IDD)**	Intervertebral disc degeneration
**(LBP)**	Low back pain
**(ECM)**	extracellular matrix
**(p38** **MAPK** **)**	p38 mitogen-activated protein kinase
**(NP)**	nucleus pulposus
**(AF)**	annulus fibrosus
**(CEP)**	cartilaginous endplate
**(COL2)**	collagen type II
**(GAG)**	glycosaminoglycan
**(MAPK)**	mitogen-activated protein kinase
**(MMPs)**	matrix metalloproteinases
**(PGE2)**	prostaglandin E2
**(SA-β-Gal)**	senescence-associated β-galactosidase
**(AP)**	autophagosomal
**(MKK)**	MAPK kinase
**(MKKK)**	MAPK kinase
**(HBO)**	hyperbaric oxygen
**(PEMF)**	pulsed electromagnetic fields
**(ER)**	endoplasmic reticulum
**(TIMP-1)**	tissue inhibitor of matrix metalloproteinase-1
**(NPMSCs)**	nucleus pulposus mesenchymal stem cells
**(HG)**	high glucose
**(MSC-CM)**	MSC conditioned medium
**(OP-1)**	osteogenic protein-1
**(RhTSG-6)**	recombinant human TNF-α induced protein 6
**(ROS)**	reactive oxygen species
**(LCM)**	laser capture microdissection
**(DHE)**	dehydrocostus lactone
**(NAC)**	N-acetyl cysteine
**(AOPP)**	Advanced oxidation protein product
**(ATM)**	ataxia telangiectasia-mutated
**(MALAT1)**	metastasis associated lung adenocarcinoma transcript 1
**(ceRNA)**	competing endogenous RNAs
**(MGF)**	Mechano growth factor
**(QUE)**	Quercetin
**(DHJSD)**	Duhuo jisheng decoction
**(3-MA)**	3-Methyladenine
**(IAPP)**	islet amyloid polypeptide
**(GM-CSF)**	granulocyte macrophage-colony stimulating factor
**(CKO)**	conditional knockout
**(MKP1/DUP1)**	mitogen-activated protein kinase phosphatase-1
**(TLR)**	tyrosine-like receptor
**(COX-2)**	cyclooxygenase-2
**(ASK1)**	Apoptosis signal-regulated kinase 1
**(Trx)**	Thioredoxin
**(TRAF2)**	TNF receptor associated factor 2
